# Addressing a missed opportunity for contraceptive use during the extended postpartum period by integrating it with infant immunization services in Sidama Region, Ethiopia: A quasi-experimental study

**DOI:** 10.1371/journal.pgph.0003236

**Published:** 2024-06-25

**Authors:** Abebaw Abeje Muluneh, Abel Gedefaw, Temesgen Kelaye, Friehiwot Sisay, Misganaw Worku, Ayalew Astatkie, Solomon Shiferaw

**Affiliations:** 1 Department of Midwifery, College of Medicine and Health Sciences, Debremarkos University, Debremarkos, Ethiopia; 2 Department of Obstetrics and Gynecology, College of Medicine and Health Sciences, Hawassa University, Hawassa, Ethiopia; 3 Research and Technology Transfer Directorate, Southern Nations, Nationalities, and People’s RegionHealth Bureau, Hawassa, Ethiopia; 4 Midwifery Services Coordination Unit, Hawassa University Comprehensive Specialized Hospital, Hawassa, Ethiopia; 5 School of Public Health, College of Medicine and Health Sciences, Hawassa University, Hawassa, Ethiopia; 6 School of Public Health, College of Medicine and Health Science, Addis Ababa University, Hawassa, Ethiopia; PLOS: Public Library of Science, UNITED STATES

## Abstract

Globally, unmet need for postpartum family planning is high. However, immunization services are among the most widely utilized health services. Establishing systematic screening, counseling, and referral systems from different contact points, particularly from EPI units may improve postpartum family planning uptake. Hence, this study aimed to assess the effect of counseling for family planning at EPI units on contraceptive uptake during the extended post-partum period. A before-and-after type of quasi-experimental study was conducted in 8 purposively selected primary health care units in Sidama region, Ethiopia. All mothers visiting the selected health facilities for infant immunization services from February 06 to August 30, 2020, were screened, counseled, and referred for family planning. A structured interviewer-administered questionnaire was used to collect data from 1421 randomly selected mothers (717 for pre-intervention and 704 post-intervention phases). EpiData version 3.1 and SPSS version 22 were used for data entry and analysis. The effect of the intervention was assessed using multivariable logistic regression analysis adjusting for the effects of potential confounders. P value < 0.05 was considered statistically significant. The contraceptive utilization rate before intervention was 72.7% with 95% CI (69.5, 75.9). It was 91.9%, 95% CI (89.8%, 93.9%) after the intervention. Utilization of contraceptive pills increased from 4.3% to 6.9%, injectables from 52.4% to 57.5%, implants from 12.8% to 22.9%, and IUCD from 3.2% to 5.0% after the intervention. After adjusting for the effect of possible confounding variables, screening, counseling, and referring mothers for family planning at infant immunization units significantly increases the contraceptive utilization rate among mothers presented for infant immunization services(AOR = 5.83, 95% CI: 4.02, 8.46). Screening, counseling, and referring mothers for family planning services at infant immunization units significantly increases postpartum contraceptive uptake. Integrating family planning messages with infant immunization services is recommended.

**Trial registration**: ClinicalTrials.gov Identifier: NCT04767139 (Registered on 23/02/2021).

## 1. Introduction

Family planning is a key and effective way of avoiding adverse maternal and perinatal outcomes by preventing unintended and closely spaced pregnancies [1]. It can be directly or indirectly used to achieve most of the Sustainable Development Goals (SDGs). Reaching all reproductive-age women with family planning who want or need it has been a global agenda for decades. However, unmet need for family planning remains high specifically in developing countries including Ethiopia [2, 3]. The Overall unmet need in urban and rural areas of Ethiopia was 7.2% and 19.1%, respectively [3]. The unmet need for family planning in Arba Minch Zuria and Hawassa Zuria districts in South Ethiopia was 41.5% and 19.1%, respectively [2, 4].

Postpartum family planning (PPFP) has an additional advantage of preventing closely spaced pregnancies over avoiding unintended pregnancies [5]. However, postpartum women are among those with the greatest unmet need for family planning services [6, 7, 10]. Most women in the extended postpartum period, the period from birth to one year (12 months), want to delay or avoid future pregnancies but many are not using any modern contraceptive method [8]. Analysis of a series of Demographic and Health Survey (DHS) data from 27 countries indicates two-thirds of women in the extended postpartum period have an unmet need for contraception [8]. An analysis of data from 17 countries illustrated that the unmet need for contraception among this population is very high, ranging from 45% to more than 80% of postpartum women [9].

Women, especially in developing countries, often wait for the resumption of menses to initiate contraceptives during the postpartum period [10–12]. Generally, nearly all of the factors associated with post-partum contraceptive uptake can be directly or indirectly resolved through comprehensive counseling [13]. Different programs and strategies have been implemented in Ethiopia to improve family planning counseling [14]. However, the coverage and quality of family planning counseling in Ethiopia are still low. Establishing systematic screening, counseling, and referral systems from different contact points, particularly from EPI units may improve family planning access and uptake in the extended perinatal period [15, 16].

Although evidence is limited, providing family planning information and/or services to postpartum women during their infants’ immunization visits may be an effective way to reach women with high unmet needs for family planning because infant immunization services are among the most widely utilized health services [17, 18].

Therefore, to use such an opportunity afforded by immunization visits to screen and counsel for contraceptive services, and to integrate postpartum family planning within infant immunization services, there is a need for additional rigorous evidence and literature. However, there is a paucity of such evidence in Ethiopia. Hence, this study was designed to assess the effectiveness of screening, counseling, and referring mothers during their infant immunization visits to improve contraceptive uptake during the extended postpartum period in Sidama region, Ethiopia.

## 2. Materials and methods

### 2.1 Study design and population

A before-and-after type of quasi-experimental study was conducted in eight purposely selected health centers in Sidama, Ethiopia from February 06 to August 30, 2020. These healthcare facilities, which are parts of the primary healthcare unit, were purposely selected from both urban and rural areas because they had relatively better case flow. In these health centers, except Yirgalem Health Center, immunization and family planning units are located side by side.

Pre and post-intervention assessment of contraceptive utilization rates among mothers visiting primary health care units for infant immunization services was done in these centers. The pre-and post-intervention comparison was made between two different groups of women. A baseline study (“before” study) was conducted before the implementation of the intervention. Following the baseline study, the intervention was implemented.

**Fig 1:**
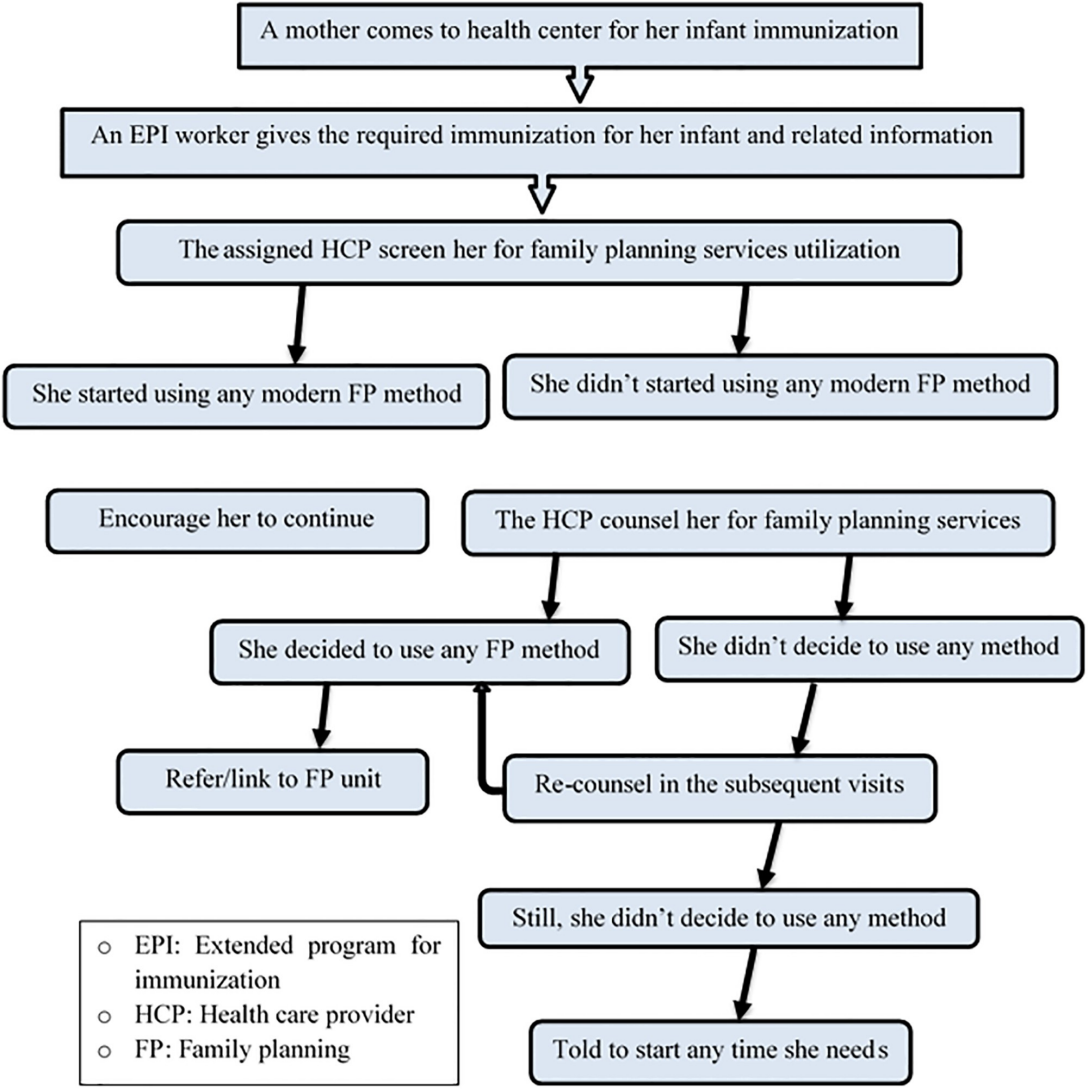
A flow chart summarizing the integration of family planning counselling with infant immunization services in Sidama region, Ethiopia, 2020.

The intervention comprised screening, counseling, and referring mothers for family planning services. All mothers who visited the health centers for infant immunization services were screened and counseled for family planning services, and those who decided to take any modern family planning method were referred/linked to family planning units. All mothers who did not decide to use were re-counseled by the next visit (Fig 1). The intervention was implemented by nurses and midwives working in the EPI and family planning units of the selected health facilities. These health care providers were provided a three-day training so that they have adequate knowledge and skills for such an activity. The training was facilitated by an Obstetrician and gynecologist, a clinical Midwifery specialist, and a BSc midwife who all have TOT (Training of Trainers) and have been providing family planning training for several years. For this training, the national postpartum contraceptive training guideline prepared by the Ethiopian Ministry of Health was used.

A laminated REDI (Rapport building, Exploration, Decision making, and Implementing the decision) family planning counseling guide was provided for each healthcare provider to be used during the training and implementation periods. REDI family planning counseling guide consists of the details of the steps to be followed during screening and counseling for family planning starting from greeting a client, exploring her knowledge about family planning and fertility plan, explanation about methods, method eligibility, encouraging her to make her own decision, identifying barriers to implement her decision … to follow up plan. Throughout the implementation period, one midwife and one nurse were assigned for screening and counseling purposes. On days when case flow was high, this number was doubled. A supervisor, from the investigators, was assigned at least twice a week to each health facility to supervise the proper implementation of the intervention as well as to solve any problematic issues.

A mother was assumed to be adequately exposed to this intervention if and only if she was counseled for family planning at least two times (on two EPI visits). Therefore, the post-intervention study was started at least two months after the initiation of the intervention because the first, second, and third EPI visits are scheduled every month, and mothers have at least two exposures for the intervention.

The primary outcome of the intervention was improved contraceptive uptake during the first year after delivery. It was calculated as the proportion of women in the first year of the postpartum period who used modern family planning methods during that period.

All women in the extended postpartum period coming to the selected health facilities for infant immunization services during the study period constituted the study population. For the pre-intervention study, all women coming to the selected health facilities at least two times during the pre-intervention assessment period were included. Whereas, women who have at least two health facility visits for their infant immunization services after the initiation of the intervention were included in the post-intervention evaluation. Mothers referred to and from the selected health facilities for infant immunization services were excluded.

### 2.2 Sampling

The sample size was calculated using OpenEpi statistical software assuming a 15% increase in the contraceptive prevalence rate during the extended postpartum period after the intervention, 80% power, 95% confidence interval (CI), a 1:1 pre and post-intervention ratio, and taking the contraceptive prevalence rate of 54.7% among mothers in the extended postpartum period in Dessie, Ethiopia [19],. Considering the 10% possible non-response rate, the sample size became 1474, 737 for each phase (before and after intervention). The sample size was proportionally distributed for each selected health center depending on the case flow. Finally, study subjects were selected using a systematic random sampling technique from each health center.

### 2.3 Data collection

Data were collected using a pretested, structured, interviewer-administered questionnaire prepared in English and translated to the local language, Amharic. The questionnaire has two parts. The first part details about sociodemographic characteristics of the participants, and the second part asks about childbirth and reproductive health issues including family planning service utilization. The same data collection tool was used for both pre and post-intervention phases.

A total of eight data collectors with at least a diploma in Midwifery or Nursing did the data collection. Two supervisors with at least an MSc/MPh supervised the data collection process.

Before the actual data collection, the questionnaire was pre-tested on 37 (5%) of the total sample size in health centers that were not included in the study, and necessary modifications were made accordingly. Throughout the course of data collection, regular meetings were held among data collectors, supervisors, and investigators. The collected data were reviewed and checked for completeness before data entry.

### 2.4 Data processing and analysis

Data were cleaned, coded, and entered into Epi data version 3.1 and exported to SPSS version 22 for analysis. The effect of the intervention was assessed using multivariable logistic regression analysis adjusting for the effects of potential confounders. The potential confounding variables listed in Tables 1 and 2 undergo bivariate logistic regression analysis, and those with a P value of < 0.25 were considered for multivariable logistic regression analyses. Multicollinearity and model fitness tests were done. Finally, statistical significance was made for P < 0.05, and an adjusted odds ratio (AOR) at a 95% confidence interval was used to examine the presence and strength of the effect of the intervention on post-partum contraceptive utilization.

**Table 1:** Socio-demographic characteristics of the participants in Sidama region, Ethiopia, 2020.

Variables		Before intervention		After intervention	
		Frequency	Percent	Frequency	Percent
Age of participants (years)	15–24	291	40.6	225	32.1
	25–34	378	52.8	421	60.1
	35–50	47	6.6	54	7.7
Religion	Orthodox	98	13.7	87	12.4
	Protestant	572	79.8	563	80.0
	Muslim	34	4.7	47	6.7
	Others	13	1.8	7	1.0
Educational status	No formal education	97	13.5	96	13.6
	Primary school	368	51.3	338	48.0
	Secondary school	137	19.1	129	18.3
	College and above	115	16.0	141	20.0
Occupation	Housewife	534	74.5	556	79.0
	Farmer	8	1.1	2	0.3
	Merchant	48	6.7	21	3.0
	Gov’t Employee	80	11.2	87	12.4
	Private organization	26	3.6	18	2.6
	Others	21	2.9	20	2.8
Residence	Urban	480	66.9	557	79.1
	Rural	237	33.1	147	20.9
Age at marriage (years)	< 18	128	18.2	96	14.3
	18–25	552	78.6	565	84.1
	> 25	22	3.1	11	1.6
Husband’s educational status	No formal education	73	10.2	56	8.0
	Primary education	254	35.4	203	28.9
	Secondary education	200	27.9	213	30.3
	College and above	190	26.5	231	32.9
Husband’s occupation	Merchant	224	31.3	228	32.7
	Farmer	145	20.3	110	15.8
	Gov’t employee	149	20.8	184	26.4
	Private organization	124	17.3	139	19.9
	Others	74	10.3	36	5.2
Distance to the nearest health facility	< 20 Minutes	354	49.4	385	54.7
	20–60 Minutes	353	49.3	307	43.6
	> 60 Minutes	9	1.3	12	1.7

**Table 2:** Childbirth and reproductive health characteristics of the participants in Sidama region, Ethiopia, 2020.

Variables		Before intervention		After intervention	
		Frequency	(%)	Frequency	(%)
Parity	1	261	36.4	218	31.0
	2–3	320	44.6	348	49.4
	4–6	126	17.6	134	19.0
	7–9	10	1.4	4	0.6
Number of alive children	1	270	37.7	227	32.2
	2–4	379	52.9	429	60.9
	≥5	68	9.5	48	6.8
ANC follow up	Yes	687	95.8	696	98.9
	No	30	4.2	8	1.1
Place of birth	Home	174	24.3	113	16.1
	Health facility	543	75.7	591	83.9
Birth assistance	Skilled	543	75.7	592	84.1
	Non-killed	174	24.3	112	15.9
Mode of delivery	Vaginal	632	88.1	605	85.9
	Abdominal	85	11.9	99	14.1
Sex of the child	Female	386	53.8	368	52.3
	Male	331	46.2	336	47.7
PNC use	Yes	303	42.3	278	39.6
	No	414	57.7	424	60.4
Postpartum period duration	<14 weeks	337	47.0	443	63.0
	≥ 14 weeks	380	53.0	260	37.0
Return of menstruation (in days)	Didn’t resume	365	52.1%	282	40.1%
	10–45	194	27.7%	204	29.0%
	46–89	123	17.5%	217	30.8%
	90–125	19	2.7%	1	0.1%
Sexual activity resumed	Yes	572	79.8%	535	76.3%
	No	145	20.2%	166	23.7%

### 2.5 Ethical considerations

Ethical clearance was obtained from the Institutional Review Board (IRB) of the College of Medicine and Health Sciences, Hawassa University. The ethical clearance paper was then presented to Hawassa town administration and health officials in Sidama to obtain official permission to undertake the research activities in the selected health centers.

After detailed explanations of the purpose and the total course of the study, written informed consent was obtained from each participant just before the actual data collection. There was no harm associated with participation in this study. Participation was based on the participant’s willingness to assure confidentiality, Personal data was not collected.

## 3. Results

Of the expected 1,474 study participants, 1,421 mothers in the extended postpartum period (717 for the pre-intervention and 704 for the post-intervention phases) took part in the study resulting in a response rate of 96.5%.

### 3.1 Sociodemographic characteristics of the participants

The participants were in the age range of 15–50 years. The mean (±standard deviation [SD]) age of the participants involved in the before and after phases of the intervention was 25.69 (±4.87) and 26.48(±4.41) years, respectively. More than two-thirds, 480 (66.9%) and 557(79.1%), of the participants in the pre and post-intervention groups, respectively, were urban residents. Even though an equal number of health centers were recruited from rural and urban areas, the case flow in urban health centers was relatively higher and this might be the reason for such discrepancy. Most, (86.4%) and 608(86.3%), of the participants in the pre and post-intervention phase had attended at least primary school. On the other hand, 534(74.5%) of the pre-intervention and 556(79.0%) of the post-intervention group were housewives in occupation. Five hundred seventy-four (81.7%) of the participants in the pre-intervention and 576 (85.7%) of post-intervention groups, respectively, had their first marital relationship after celebrating their 18^th^ birthday (Table 1).

### 3.2 Childbirth and reproductive health issues

About two-thirds, 456(63.6%) of the participants in the before-intervention phase and 486(69.0%) in after intervention phase were multiparous. The mean (±SD) number of alive children each participant had in the before and after intervention phases was 2.29 (±1.45) and 2.32(±1.30), and 28 (3.9%) and 27(3.8%), respectively had experienced child death (Table 2).

### 3.3 Family planning utilization during the extended post-partum period

**Fig 2:**
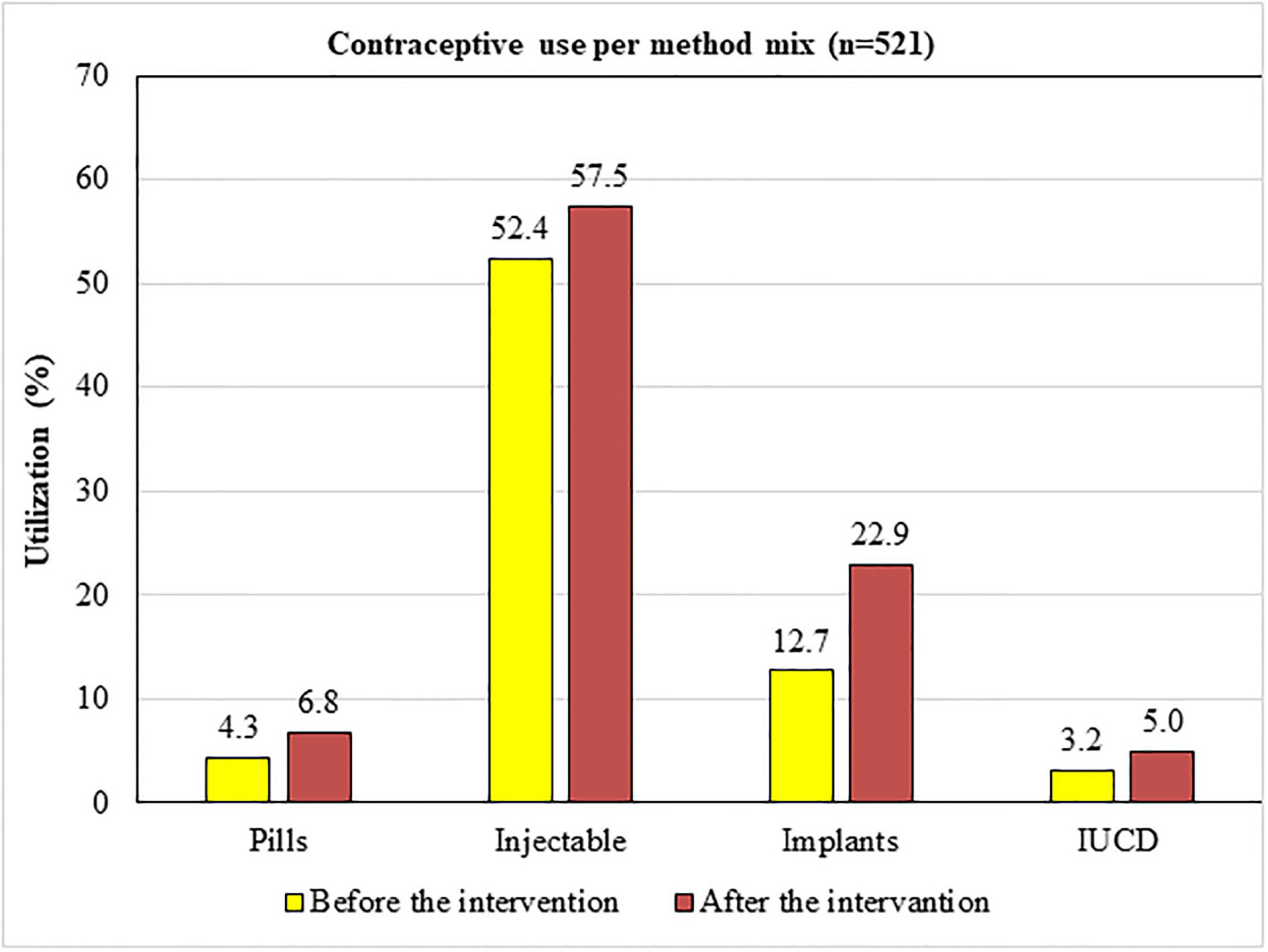
Contraceptive utilization by method mix among mothers presented to EPI units in Sidama Region, Ethiopia, 2020.

Five hundred twenty-one (72.7%), with a 95% CI of (69.5%, 75.9%), of the mothers in the pre-intervention group, and 647 (91.9%) with a 95% CI of (89.8%, 93.9%) of the mothers in the post-intervention group have been using modern Contraceptive methods. This implies that the Contraceptive utilization rate among mothers presented to health centers for infant immunization increased by 26.4% after the intervention (counseling at immunization units) was started. The increase in the utilization of long-acting contraceptive methods (74.8%) was much higher than that of short-acting methods (13.2%) (Fig 2).

### 3.4 Effect of counseling for family planning on postpartum contraceptive uptake

Age of the participants, age of the participants at marriage, antenatal follow-up during the recent pregnancy, place of birth, mode of delivery, length of the postpartum period, sexual activity resumption, and menses resumption were among the predictor variables which had a statistically significant association with postpartum contraceptive utilization on bivariate analysis. After adjusting for these variables in the multivariable model, counseling mothers for family planning at EPI units and linking or referring to family planning services was found to have a statistically significant association with postpartum contraceptive utilization. The odds of using postpartum contraceptives after the initiation of the intervention was nearly 6 times higher than that before the intervention (AOR: 5.83; 95% CI: 4.02, 8.46) (Table 3).

**Table 3:** Multivariable analysis to test the effect of counseling mothers at EPI units on postpartum contraceptive utilization in Sidama region, Ethiopia, 2020.

Variables		FP use		COR (95% CI)	AOR (95% CI)
		Yes	No		
Data: Before/after the intervention	Before	521	196	1.00	1.00
	After	647	57	4.27(3.11, 5.86)	5.83(4.02, 8.46)**
Age of the participants in years (n = 1416)	15–24	435	81	1.23(0.92, 1.66)	1.17(0.82, 1.66)
	25–34	650	149	1.00	1.00
	35–50	78	23	0.78(0.47, 1.28)	0.76(0.42, 1.37)
Age of the participants at marriage in years (n = 1374)	< 18	183	41	0.96(0.66, 1.39)	0.97(0.63, 1.50)
	18–25	920	197	1.00	1.00
	> 25	19	14	0.29(0.14, 0.59)	0.43(0.19, 1.02)
ANC use	Yes	1146	237	1.00	1.00
	No	22	16	0.28(0.15, 0.55)	0.43(0.19, 0.97)*
Place of birth	At home	220	67	1.00	1.00
	Health facility	948	186	1.55(1.13, 2.13)	1.45(0.98, 2.14)
Mode of delivery	Vaginal	1028	209	1.00	1.00
	Abdominal	140	44	0.65(0.45, 0.94)	0.70(0.45, 1.11)
Postpartum period duration (in weeks) (n = 1420)	10 to 14	625	155	1.00	1.00
	≥ 14	542	98	1.37(1.04, 1.81)	1.37(0.99, 1.91)
Menses resume	Yes	695	79	1.00	1.00
	No	473	174	0.31(0.23, 0.41)	0.16(0.12, 0.23)**
Sexual activity resume (n = 1418)	Yes	980	127	1.00	1.00
	No	186	125	0.19(0.14, 0.26)	0.49(0.35, 0.68)**

* P< 0.05

** P< 0.001

## 4. Discussion

A statistically significant increase in postpartum contraceptive utilization rate was observed among mothers who presented for infant immunization services following the implementation of counseling services for family planning at EPI units. A 19.2% increase in contraceptive utilization rate was documented after the intervention was initiated.

This is consistent with the findings in a clustered randomized controlled trial in Rwanda [20], in which an increased post-partum family planning utilization was observed by integrating family planning and EPI Services. This is probably because counseling provides the opportunity for women to be educated about postpartum return to fertility and encouraged to space or limit pregnancy by using effective contraceptives. Using infant immunization units gives an additional opportunity to reach more mothers who want to use family planning in the postpartum period.

Family planning counseling increases the intention to use postpartum contraceptives [21]. Women who received counseling are more likely to use effective contraceptives [22]. Counseling also provides factual information on types of contraceptive methods, safety, sources of supply, and management of side effects and increases awareness and acceptance of contraception by encouraging individuals to look to a better future and promoting family planning as one means to that end [23]. A statistically significant association between utilization of postpartum family planning services and counseling on fertility intention and family planning was also reported in Ntchisi District Hospital, Malawi [15] and Lagos University Teaching Hospital, Nigeria [21]. Poor and/or inadequate client counseling was reported to have a negative impact on postpartum contraceptive utilization in studies conducted in Gondar, North Western Ethiopia [24]. Adequately counseled mothers are more likely to use contraception [25].

Many postpartum women in Ethiopia wait for the resumption of menstrual periods to start using contraception [26, 27]. However, they are at risk of unintended pregnancy. Hence, counseling women about postpartum return of fertility and encouraging them to use an effective contraceptive method is invaluable [20] and requires focusing attention to achieve optimal birth spacing [28, 29].

Family planning counseling enhances the quality of women’s contraceptive decision-making as it allows women and providers to discuss challenges and preconditions required to come up with shared decision-making [29] and helps clients balance the benefits and disadvantages of the available family planning methods [30]. Moreover, it is also recommended by World Health Organization (WHO) clients to make informed decisions because counseling is considered an important part of informed choice [31].

Counseling for family planning is a means of promoting postpartum contraceptive services in Africa and Asia where awareness and knowledge of fertility return is a major concern [32]. As counseling equips women with factual information on the types, safety, effectiveness, and side effects of contraceptive methods [23, 29, 33], clients are more satisfied with their method of choice [34]. ’Contraceptive discontinuation as a result of side effects can be minimized or avoided by counseling [25, 26]. A literature review concluded a fourfold increased likelihood of continuing with contraceptive methods when mothers are counseled for side effects [25].

Significantly increased postpartum modern contraceptive use with family planning counseling during postnatal care was observed in Gida Ayana district, West Ethiopia [35], Aksum [14], and Northwest Ethiopia [36, 37]. A fivefold higher likelihood of opting for long-acting reversible contraceptives was also reported among mothers who received counseling services during the postpartum period in Durame, South Ethiopia [38]. A Systematic review and meta-analysis in Ethiopia also concluded that antenatal and postnatal care services were significantly associated with postpartum contraceptive use [37].

Counseling for family planning during other maternal health care services including prenatal care and delivery are also key contact points to promote postpartum modern contraception [39]. Significantly increased postpartum contraceptive utilization with family planning counseling during prenatal care was observed in Ethiopia [14, 36, 40] and Mexico [41]. A higher likelihood of timely initiation of postpartum contraceptives was also reported among mothers getting family planning counseling at delivery in Pawe district, northwest Ethiopia [42], and Durame [38]. Moreover, a longitudinal study in Ethiopia concluded that integration of postpartum family planning counseling into postnatal care services is an effective means to increase postpartum contraceptive uptake [33].

Readers, please note that since the health facilities included in this study were selected purposively, selection bias may be introduced. The study also lacked a control group and covered a small area.

## 5. Conclusion

In this study, screening, counseling, and referring mothers for family planning services at infant immunization units was found to significantly enhance contraceptive uptake during the extended postpartum period. The effect of the intervention was more pronounced in increasing long-acting reversible contraceptive methods than that of short-acting ones. Screening, counseling, and referring all mothers visiting health facilities for their infant immunization services to family planning services is recommended.

## Supporting information

S1 Checklist STROBE checklist. (DOCX)

S2 Checklist TREND statement checklist. (PDF)

S1 Table CONSORT table. (DOCX)

S1 Data (SAV)
