# Correction: Addressing a missed opportunity for contraceptive use during the extended postpartum period by integrating it with infant immunization services in Sidama Region, Ethiopia: A quasi-experimental study

**DOI:** 10.1371/journal.pgph.0004101

**Published:** 2024-12-12

**Authors:** Abebaw Abeje Muluneh, Abel Gedefaw, Temesgen Kelaye, Friehiwot Sisay, Misganaw Worku, Ayalew Astatkie, Solomon Shiferaw

There is an error in affiliation 6 for author Solomon Shiferaw. The correct affiliation 6 is: School of Public Health, Addis Ababa University, Ethiopia.
